# Global wheat planting suitability under the 1.5°C and 2°C warming targets

**DOI:** 10.3389/fpls.2024.1410388

**Published:** 2024-06-17

**Authors:** Xi Guo, Puying Zhang, Yaojie Yue

**Affiliations:** Key Laboratory of Environmental Change and Natural Disaster of Chinese Ministry of Education, Faculty of Geographical Science, Beijing Normal University, Beijing, China

**Keywords:** climate change, 1.5°C and 2°C warming targets, global wheat planting suitability, Maxent, robust analysis

## Abstract

The potential distribution of crops will be impacted by climate change, but there is limited research on potential wheat distributions under specific global warming targets. This study employed the Maxent model to predict the potential distribution of wheat under the 1.5°C and 2°C warming targets based on data from the Inter-Sectoral Impact Model Intercomparison Project (ISI-MIP) multimodel ensemble, and the effect of global warming on wheat planting suitability was analyzed. Our results indicated global warming would significantly change wheat planting suitability. Over half of the areas experienced changes in wheat planting suitability under two warming targets, and the effect became more pronounced with increasing temperatures. Additionally, global warming might promote wheat planting in more regions. The area with an increase in wheat planting suitability was observed to be 9% higher than those experiencing a decrease on average. Moreover, global warming could exacerbate the disparity between global wheat supply and demand in countries/regions. Traditional wheat-producing countries/regions are poised to benefit from the warming effects of climate change, while less developed and wheat import-dependent countries/regions may face greater challenges in achieving wheat self-sufficiency. To address this potential challenge, the promotion and inter-regional exchange of agronomic technologies, and the development of more rational trade standards are urgently needed. Since socioeconomic factors have a significant impact on wheat cultivation, further investigation is required to determine how the wheat planting distribution may change in the future under the combined impact of climate change, supply-demand relationship, and policy.

## 1 Introduction

As the most ambitious effort to reduce the risks and impacts caused by climate change thus far, the United Nations Framework Convention on Climate Change (UNFCCC) Paris Agreement proposed the aim of “holding the increase in global average temperature to well below 2°C above preindustrial levels and pursuing efforts to limit the temperature increase to 1.5°C” in 2015 (UNFCCC, 2015). This has raised a crucial research task to improve understanding of the difference in risk between the 1.5°C target and the 2°C target (Klutse et al., 2018). For example, the effects of this difference on crop yield have been examined, which suggests that a 2°C target increase would have more detrimental impacts on global food production and security (Liu et al., 2019). However, most of the existing studies on the impact of agricultural sectors under the climate change scenarios, including 1.5°C and 2°C warming targets seldom consider the dynamic change of crop distribution. Though researchers have also demonstrated that climate change will affect crop areas and, consequently, impact food production (Yue et al., 2019; Su et al., 2021, 2023). This ignoring of crop area offers an incomplete picture of crop production because of the potential for compensation or compounding of yield impacted by changes in harvested area (Lesk et al., 2016). Therefore, it is very necessary to investigate disparities in wheat distribution quantitatively under the 1.5°C and 2°C targets, because of the scarcity of such studies.

There are currently four main methods for obtaining crop areas. Administrative statistics are a traditional way to derive information on crop areas. It obtains information on crop distributions by collecting crop statistical information reported from subordinate administrative units (Liu and Li, 2006; Tan et al., 2014). For example, Liu and Li (2006) extracted information from the cost-income data of farm produce and the China Agricultural Yearbook and then studied the regional differences in the changes in agricultural land use in China during the period 1980–2002. However, due to the limitations of unit size and shape, administrative statistics cannot be directly applied to crop spatial and temporal dynamics analysis, let alone analyse the impact of climate change on crop area (Liu et al., 2013; Tan et al., 2014).

The development of satellite remote sensing provides a new technical method for analysing the temporal and spatial variations in crop area (Xiao et al., 2005; Zhang et al., 2017). This method often calculates indices and then uses an algorithm to classify the indices to extract crop area information (Teluguntla et al., 2015; Han et al., 2021, 2022). For instance, Han et al. (2021) generated the spatiotemporal trends in annual paddy rice planting areas and cropping intensity in Asia from 2000 to 2020 based on remote sensing data from multiple sources. Although remote sensing can be used to extract information with high accuracy, crop information at the pixel level is still difficult to obtain over large scales (Verburg et al., 2011; Tan et al., 2014). Moreover, these studies are often more concerned with spatial and temporal variations in crop area, considering climate change as a given condition rather than a driving force (Liu et al., 2015). Consequently, the crop area under future climate change has been relatively unexplored.

The third type of method analyses the shifting of potential crop boundaries using environmental constraints on growing crops (Sun et al., 2015; Liu et al., 2021; Su et al., 2023). For example, Yang et al. (2015) analysed the impacts of climate change on the northern limits and crop planting areas of multiple cropping systems in China, in which the northern limits for different cropping systems were distinguished by defining a certain threshold of annual accumulated temperature above 0°C. Directly constraining the distribution of crops with environmental factors is conducive to analysing the impact of climate change on crop area. However, most of the studies considered only the constraints of meteorological factors on crop growth, ignoring the influence of soil, even though it plays an important role in crop planting (Mohammed et al., 2016). The prediction based on the planting boundary did not achieve satisfactory accuracy, the spatial heterogeneity inside the cropland was ignored, and climate change consequences for agriculture differed depending on geography (Piao et al., 2010; Tchebakova et al., 2011). Moreover, it is difficult to perform accuracy tests because there are no crop samples to provide *a priori* information.

Species distribution models (SDMs) constitute the fourth methodological approach utilized for predicting potential crop distributions, assessing the potential distribution of a species through analysis of its presence samples and the environmental variables influencing its distribution (Phillips and Dudík, 2008; Elith et al., 2011). The Maxent model is particularly noteworthy among various SDMs due to its ability to achieve favorable results using limited species samples and corresponding environmental variables (Kramer Schadt et al., 2013; Yackulic et al., 2013). The outputs of the Maxent model, which indicate the probability of crop presence, effectively capture spatial heterogeneity in crop suitability (Yue et al., 2019; Guo et al., 2024), making it widely applicable at various scales for obtaining the probability of species presence in different regions (Boria et al., 2014; Angelieri et al., 2016; Khan et al., 2022; Yang et al., 2022).

However, there are still some problems to be solved when predicting crop area using SDMs, including the Maxent model. Most studies focused on specific time periods under particular emission scenarios, e.g., Representative Concentration Pathway (RCPs) (Liu et al., 2016; Shabani and Kotey, 2016), while studies that assessed the impacts of specific targets, such as 1.5°C and 2°C, on crop distribution were rarely explored. In addition, datasets estimated by global climate models have been widely used when assessing the impact of climate change. However, studies have shown that the uncertainty in climate projections should be considered when using General Circulation Models (GCMs) (Smith et al., 2009), and a multimodel ensemble is more reasonable than individual model results (Tebaldi and Knutti, 2007; Xu and Xu, 2012). Therefore, regarding potential distribution as an important indicator to measure crop area and wheat as the object, and motivated by the need for a better understanding of the difference in future global wheat distribution between 1.5°C and 2°C target, the objectives of this study are to (1) employ the Maxent model to estimate global wheat distribution under 1.5°C and 2°C target; (2) quantify the difference in potential global wheat planting arrangements under 1.5°C and 2°C target; and (3) assess the reliability of wheat planting suitability predicted under different climate models and RCP scenarios.

## 2 Materials and methods

### 2.1 Research framework

We structured the current study as follows: (1) First, we defined time periods for reaching various global warming targets (Section 2.2); (2) Second, we selected environmental factors that impact wheat cultivation and representative occurrence points for global wheat distribution (**Supplementary Methods**); (3) Third, we trained the Maxent model and predicted the potential distribution of global wheat under 1.5°C and 2°C warming targets (Section 2.3); (4) Finally, we evaluated the reliability of changes in wheat planting suitability under different RCPs scenarios (Section 2.4).

### 2.2 Definition of global warming targets

Global warming targets can be defined as the average global warming level compared to a specific baseline period (Klutse et al., 2018). Different definitions and terms for targets appear in the literature. However, all are compared with a preindustrial baseline (Nikulin et al., 2018) and often use an averaged window period with varying window sizes, e.g., 15, 20 or 30 years (Vautard et al., 2014; Mitchell et al., 2016; Roudier et al., 2016; Donnelly et al., 2017; Karmalkar and Bradley, 2017; King et al., 2017; Mba et al., 2018; Nikulin et al., 2018).

In the present study, we selected 1986–2005 as the baseline period, which has been widely used in assessing the impact of 1.5°C and 2°C global warming (Mohammed et al., 2016; Schleussner et al., 2016; Chen et al., 2017; Liu et al., 2018). This period was 0.6°C warmer than the preindustrial period (1850–1900) (Alexander et al., 2013). Therefore, warming of 0.9°C and 1.4°C above 1986–2005 corresponds to the internationally accepted thresholds of 1.5°C and 2°C above preindustrial levels. The timing of targets was defined as the first time the 30-year moving averages of global temperature were above 1.5°C or 2°C compared to preindustrial temperatures. Such 30-year periods are commonly used to represent the climate at the respective time (Vautard et al., 2014; Roudier et al., 2016; Donnelly et al., 2017; Mba et al., 2018; Nikulin et al., 2018), as they can limit the small trends within the period and catch the interannual and decadal variability simultaneously (Pfeifer et al., 2015).

We used data from the ISI-MIP multimodel ensemble to assess the impacts of climate change on the distribution and planting suitability of wheat. The ISI-MIP GCM outputs have been bias-corrected through a trend-preserving bias-correction method (Hempel et al., 2013), which has been widely applied to drive global impact models for assessing risks under climate change at global and regional scales (Chen et al., 2017; Dottori et al., 2018; Liu and Sun, 2019). The ensemble comprises five GCMs, namely, GFDL-ESM2M, HadGEM2-ES, IPSL-CM5A-LR, MIROC-ESM-CHEM, and NorESM1-M (abbreviated as GFDL, HAD, IPSL, MIR, and NOR, respectively).

There are 4 RCPs, among which RCP2.6 represents a stringent mitigation scenario. The RCP4.5 and RCP6.0 scenarios represent two intermediate scenarios, and RCP8.5 represents a scenario with very high GHG emissions (IPCC, 2014). The four RCPs span the range of radiative forcing scenarios in the published literature (Moss et al., 2010). However, many of the simulations do not reach the 2°C level under the RCP2.6 scenario (Vautard et al., 2014; Liu et al., 2018; Nikulin et al., 2018; Ruane et al., 2018), and transient simulations from multiple GCMs at higher greenhouse emissions could be analysed. Moreover, RCP4.5 contains the vast majority of the scenarios assessed in AR4, while the number of studies corresponding to RCP6.0 is relatively low (Vuuren et al., 2011). Based on the above, these studies chosen the RCP4.5 and RCP8.5 scenarios (Schleussner et al., 2016; King and Karoly, 2017; Liu et al., 2019).

### 2.3 Maxent model application and validation

Estimating potential wheat distribution using the Maxent model entails three essential steps: the identification of key environmental variables influencing wheat distribution, the selection of sample points representing wheat presence for training the Maxent model from a known database, and the training and validation of the Maxent model to ensure accuracy. Finally, the validated Maxent model was utilized to project wheat distribution under 1.5°C and 2°C targets.

Initially, a comprehensive wheat planting suitability estimation index system including climate and soil factors was established (Yue et al., 2019; Guo et al., 2024). Environmental variables such as temperature, precipitation, pH, drainage, soil texture, depth, and slope were used to assess wheat growth. Subsequently, we selected the wheat-harvested area fractions (Monfreda et al., 2008) as the primary source for determining the occurrence points of wheat distribution. The wheat-harvested area fractions are part of the global acreage and yield database for 175 different crops, which was compiled by collecting agricultural census data and survey information from various political units in 206 countries. Finally, we randomly selected a total of 15,500 samples representing approximately 5% of all wheat presence grids, to train the Maxent model. The selection process of environmental variables and training samples is described in detail in the **Supplementary Methods**.

We utilized baseline samples and environmental variables to train the Maxent model. In this procedure, parameters were set as follows: the training set consisted of 75% of these samples and the test set was the remaining 25%; the logistic output format (between 0–1) was chosen to represent the probability of species presence; the jackknife analysis, which created a model with the remaining variables after excluding variables in turn, was chosen to measure variable relative importance; and we retained defaults for all other parameters.

The Maxent model was verified by the receiver operating characteristic (ROC) curve and our sampling method was employed to validate the reliability of the prediction accuracy through fivefold cross-validation (Phillips and Dudík, 2008). Moreover, a scatter plot compared the Maxent predictions with FWHA results to assess the precision of our wheat distribution prediction. Finally, we evaluated spatial congruity and rationality by superimposing Maxent predictions with the FWHA, and the integrated map of FWHA and Wheat harvested area established using Spatial Production Allocation Model (SPAM) (You et al., 2014). We argue that the performance of the Maxent outputs can be proven successfully using these approaches. Then, we employed Maxent model to predict wheat planting suitability under different targets. With the climate variables under the 1.5°C and 2°C targets, 10 sets of potential planting distribution data (2RCPs×5GCMs) were obtained for each warming target, and we averaged these data to obtain the potential suitability of wheat at the particular warming target. Based on the definition of uncertainty in IPCC, four suitability levels (**Supplementary Table S2.1**) are established for wheat habitat (Yue et al., 2019).

Pairwise comparisons were used to calculate the predicted changes in wheat distribution due to different targets and the comparison among baseline, 2°C and 1.5°C. The spatial maps are generated based on the suitability change values, which range from -1 to 1. Also, the examination of the remainder value distribution, accomplished by plotting a probability density function curve, and categorizing the changes of wheat suitability into three levels (**Supplementary Table S2.2**).

### 2.4 Assessing the reliability of the wheat planting suitability prediction

To characterize how the global projections of wheat planting suitability behave under different GCMs and RCP scenarios, we assessed the reliability of the dynamic changes in wheat cultivation from multiple models. We obtained dynamic changes through pairwise comparisons, comparing the predicted distribution of wheat for different GCMs and RCP scenarios under two targets with the reference data and then assessed reliability using the following indicators.

First, we assessed the robustness of the dynamic changes. When evaluating the robustness of a certain change, there are many definitions in previous studies (Dosio and Fischer, 2018). Here, we estimate whether the change in wheat planting suitability is robust through the model agreement and the signal-to-noise ratio (SNR, i.e., the ratio of the mean to the standard deviation of the ensemble of climate change signals), which is the same as in Klutse et al. (2018); Nikulin et al. (2018) and Maúre et al. (2018). In the present study, the changes were regarded as consistent only when more than 80% of the model simulations agree on the sign, and an SNR equal to or larger than one represents the strength of the change signal.

Second, we quantified the change in wheat planting suitability by using probability density functions (PDFs) and cumulative distribution functions (CDFs), which could also reflect the impacts and uncertainties of different models on outcomes. The value of y corresponding to x represents the cumulative grid number fraction exhibiting a certain x change in suitability in the PDFs or CDFs.

Finally, a separate set of significance tests was performed across all the GCMs and RCPs using the Friedman test. The Friedman test is a nonparametric counterpart and does not assume a normal distribution for the samples. It possesses modest statistical power of the sign test for nonnormal distributions (Zimmerman and Zumbo, 1993).

### 2.5 Data source

The data employed are shown in **Supplementary Table S2.3**. To match the resolution of the ISI-MIP data (0.5°×0.5°), all data has been converted or resampled. The world maps were created using the Robinson projection, and when it was necessary to calculate the area, we used the cylindrical equal area as the projection.

## 3 Results

### 3.1 Timing of 1.5°C and 2°C global warming targets

The median year for reaching 1.5°C lies between 2020 and 2027 and is estimated to be approximately 2021, while the corresponding median time for reaching 2°C is 10–15 years later and centred at approximately 2032 (**Table 1**). There is a significant difference in the projected time period for 2°C compared to the 1.5°C target, regardless of whether one compares between the two RCPs or among the five GCMs. The projected gap between 1.5°C and 2°C is at a maximum of 7 years and 33 years, respectively. Similarly, the variance in the centre years that reached 1.5°C was 7.61, whereas it was 78.67 for 2°C. Such a difference could result from the very similar emission trajectories and radiative forcing to the 2030s among RCP scenarios (Nikulin et al., 2018). In contrast, the gap between the two temperature thresholds is smaller under the RCP8.5 scenario when compared to the RCP4.5 scenario. This points to an increased accumulation of greenhouse gases under the RCP8.5 scenario. It should be noted that the results from GFDL showed a later threshold, which indicated lower climate sensitivity than other climate models (Ruane et al., 2018).

**Table 1:** The time periods for reaching 1.5°C and 2°C warming targets above preindustrial levels.

Climate models	RCP scenarios	1.5°C	2°C
GFDL-ESM2M	RCP4.5	2013–2042	2041–2070
	RCP8.5	2012–2041	2025–2054
HadGEM2-ES	RCP4.5	2006–2035	2014–2043
	RCP8.5	2006–2035	2008–2037
IPSL-CM5A-LR	RCP4.5	2008–2037	2021–2050
	RCP8.5	2006–2035	2016–2045
MIROC-ESM-CHEM	RCP4.5	2006–2035	2015–2044
	RCP8.5	2006–2035	2011–2040
NorESM1-M	RCP4.5	2012–2041	2028–2057
	RCP8.5	2008–2037	2020–2049

### 3.2 The global distribution and dynamic of wheat at the 1.5°C and 2°C targets

#### 3.2.1 Calculation of the preallocated shares of wheat cultivation area

**Figure 1:**
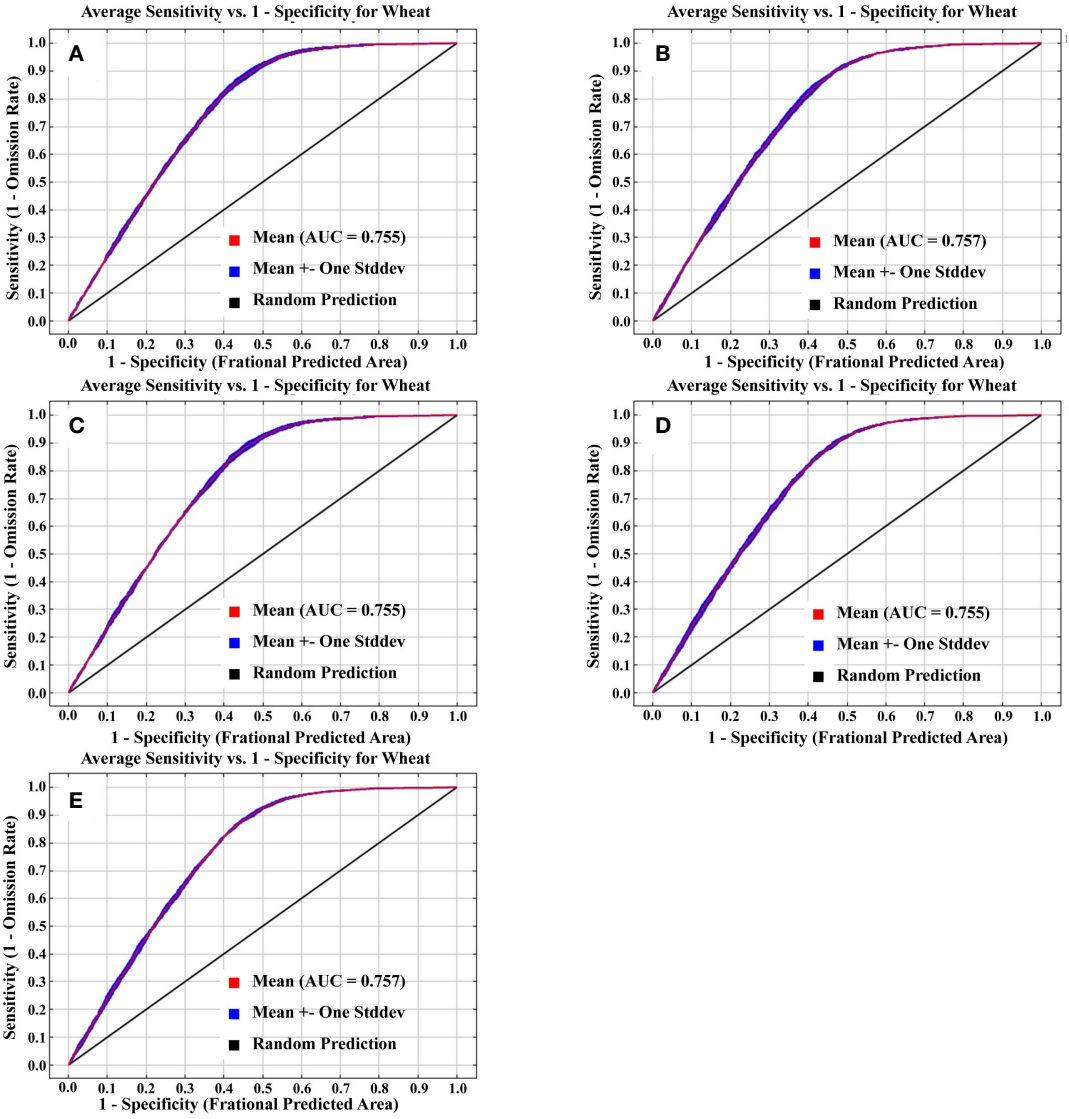
Receiver operating characteristic (ROC) curve of fivefold cross-validation for different climate models [**(A)** GFDL-ESM2M, **(B)** HadGEM2-EM, **(C)** IPSL-CM5A-LR, **(D)** MIROC-ESM-CHEM, **(E)** NorESM1-M].

The performance of different models in predicting the potential wheat planting distribution resulted in very little difference (maximum 0.002), with all predictions were above 0.75 (**Figure 1**). Moreover, the mean AUC value of the fivefold cross-validation was larger than 0.755, and the standard deviation was smaller than 0.004 (**Figure 1**), demonstrating the model’s robustness in the face of sample variations. Therefore, we argue that the predictions obtained by the Maxent model in different models have high accuracy and can predict the distribution of potential planting suitability of wheat.

The correlation between the Maxent model’s predicted suitability across different GCMs and the validation data (FWHA) was assessed through scatter plots (**Supplementary Figure S1.1**). The result indicated that the Maxent model’s forecasted land suitability for wheat cultivation was in agreement with the validation data, as the majority of the points are located near the intersection between the two median lines. Also, there is a strong alignment between the Maxent model’s projected wheat cultivation suitability and the validation data, as most estimated result points cluster close to the intersection of the two median lines.

**Figure 2:**
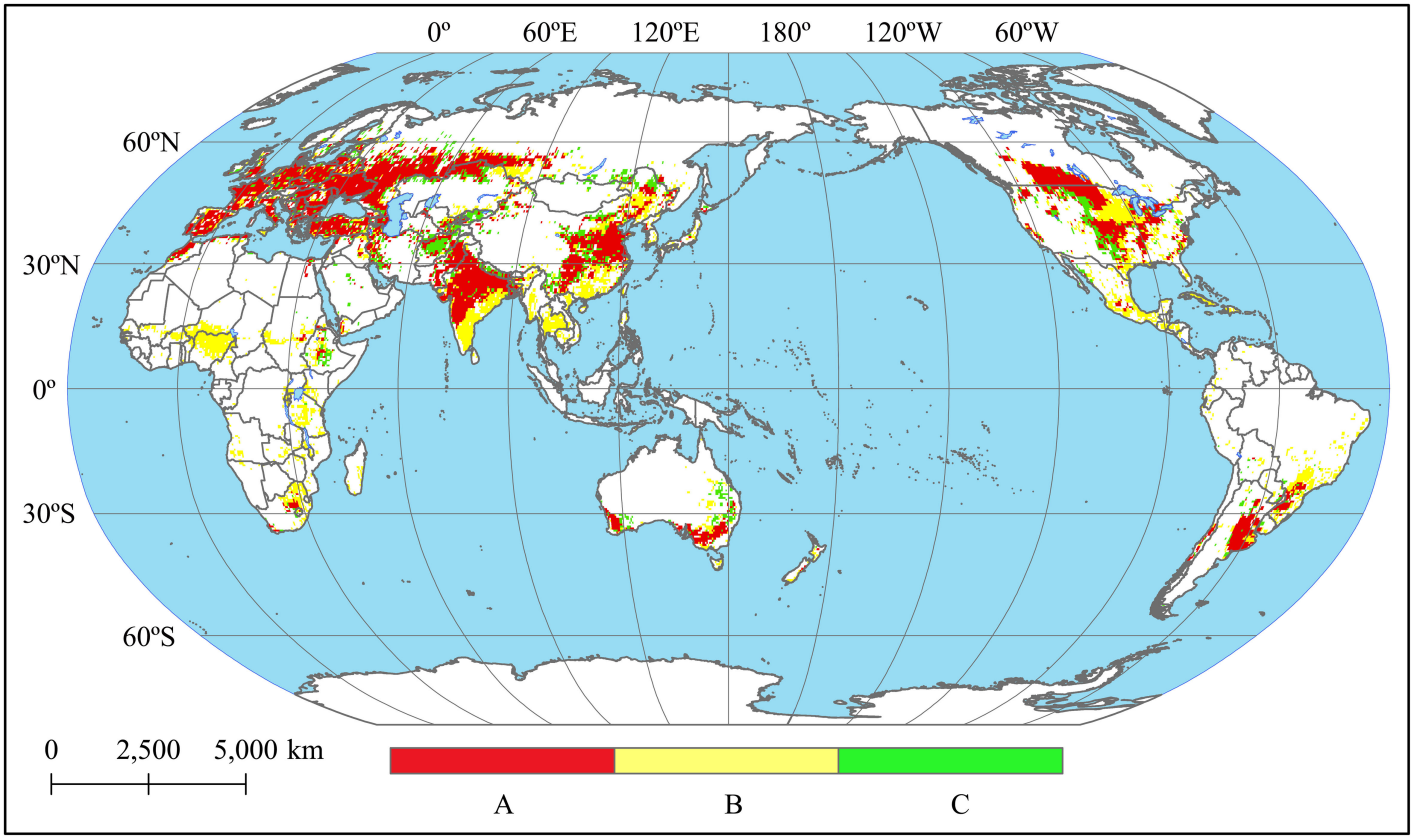
Spatial consistency between the predicted wheat planting suitability and the FWHA [**(A)** the region where wheat was both harvested and accurately predicted; **(B)** the zone showed minimal or no wheat harvesting but was expected to be suitable for wheat planting; **(C)** the region where wheat was harvested but not anticipated].

In the overlaid map depicting projected land suitability for wheat cultivation and FWHA (**Figure 2**), approximately 76% of FWHA was associated with the region where wheat was both harvested and accurately predicted (Zone A). Another 24% of FWHA fell into the category of the region where wheat was harvested but not anticipated (Zone C). The wheat harvest proportions within Zone C were notably low, suggesting these areas aren’t primary wheat production regions. Moreover, nearly 42% of the predicted land suitability for cultivating wheat was found in the region where minimal or no wheat harvesting was observed (Zone B). The validity of the results for Zone B was further examined by SPAM (**Supplementary Figure S1.2**) and the integrated (FWHA and SPAM) wheat distribution map (**Supplementary Figure S1.3**). This examination indicated Zone B was mainly located in countries or areas that are not suitable for wheat cultivation or where wheat is not a major food crop. Given that the Maxent model’s results represent potential wheat planting areas, they were expected to encompass a larger area than the actual harvested region. Therefore, the extent of the predicted land suitability for planting wheat can be considered reasonable.

#### 3.2.2 Potential wheat distribution under 1.5°C and 2°C warming targets

**Figure 3:**
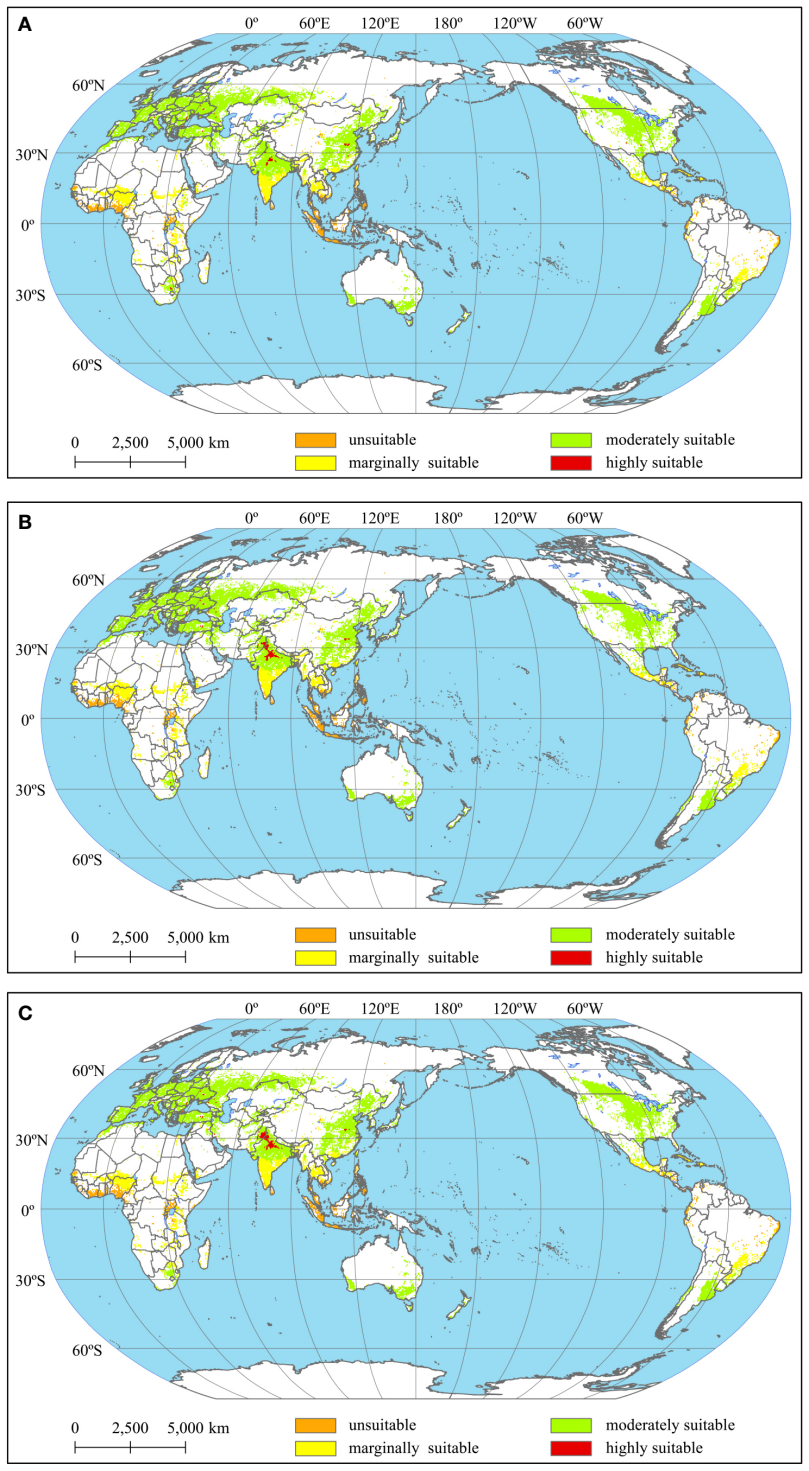
Distribution of suitability levels of wheat for different climate warming targets [**(A)** baseline period, **(B)** 1.5°C warming target, **(C)** 2°C warming target].

**Figures 3A–C** show the spatial distribution of potential land suitability levels for wheat cultivation under the baseline period, 1.5°C and 2°C targets, respectively. Compared with baseline period, the spatial pattern and potential distribution of wheat planting suitability under different targets showed an almost equivalent pattern (**Figure 3**). Highly suitable areas were found mainly in Pakistan and India. Regions displaying moderate suitability were predominantly found in central India, China, south-central Canada, central United States, southeastern Australia, Argentina, Turkey, and Europe. The marginally eligible areas were mainly in southern India, east-central Africa, and southern Mexico. The areas with marginal suitability were primarily located in southern India, east-central Africa, southern Mexico, and southern Brazil. Areas unsuitable for wheat cultivation were predominantly situated in central Africa, Indonesia and Malaysia.

**Supplementary Table S2.4** shows the proportions of area in different potential wheat suitability levels for the baseline period and for targets of 1.5°C and 2°C. The moderately suitable level occupies the largest portion of the area, approximately 65% of the total area, while the area in the highly suitable level is less than 1.5%. Compared with the baseline period, the proportion of area in the unsuitable and highly suitable levels increases, and the proportion of area in the marginally level and moderately suitable level decreases under future warming targets. Under the 1.5°C and 2°C warming targets, the area in the highly suitable level increases by 72% and 95%, respectively, compared to the baseline. Conversely, there is only a marginal expansion of areas in the unsuitable, averaging around 6% in comparison to the baseline area. Overall, no substantial evidence suggests that temperature rise significantly diminishes the suitability of wheat cultivation. On the contrary, global warming will likely enhance the wheat planting suitability in more regions. Those regions are primarily situated in frigid high latitudes, whereas wheat cultivation in warmer areas will be adversely impacted by the consequences of climate warming. These notable trends were discussed in Section 4.1 in detail.

#### 3.2.3 Effects of warming on the potential planting suitability of wheat

According to **Supplementary Table S2.5**, over half of the area with change in wheat planting suitability under two warming targets, and this change becomes more pronounced with increasing temperature. Specifically, the area where wheat planting suitability has changed under 2°C warming target was 11% higher than in the 1.5°C warming target. In two warming targets, the area with an increase in wheat planting suitability is greater than those with a decrease. Specifically, under 1.5°C and 2°C warming targets, the area where wheat planting suitability increase is 9% and 10% higher than those witnessing a decrease, respectively (**Supplementary Table S2.5**). This indicated that global warming would significantly change wheat planting suitability, and rather than wheat planting suitability decrease, future warming would favour growing wheat in more areas.

**Figure 4:**
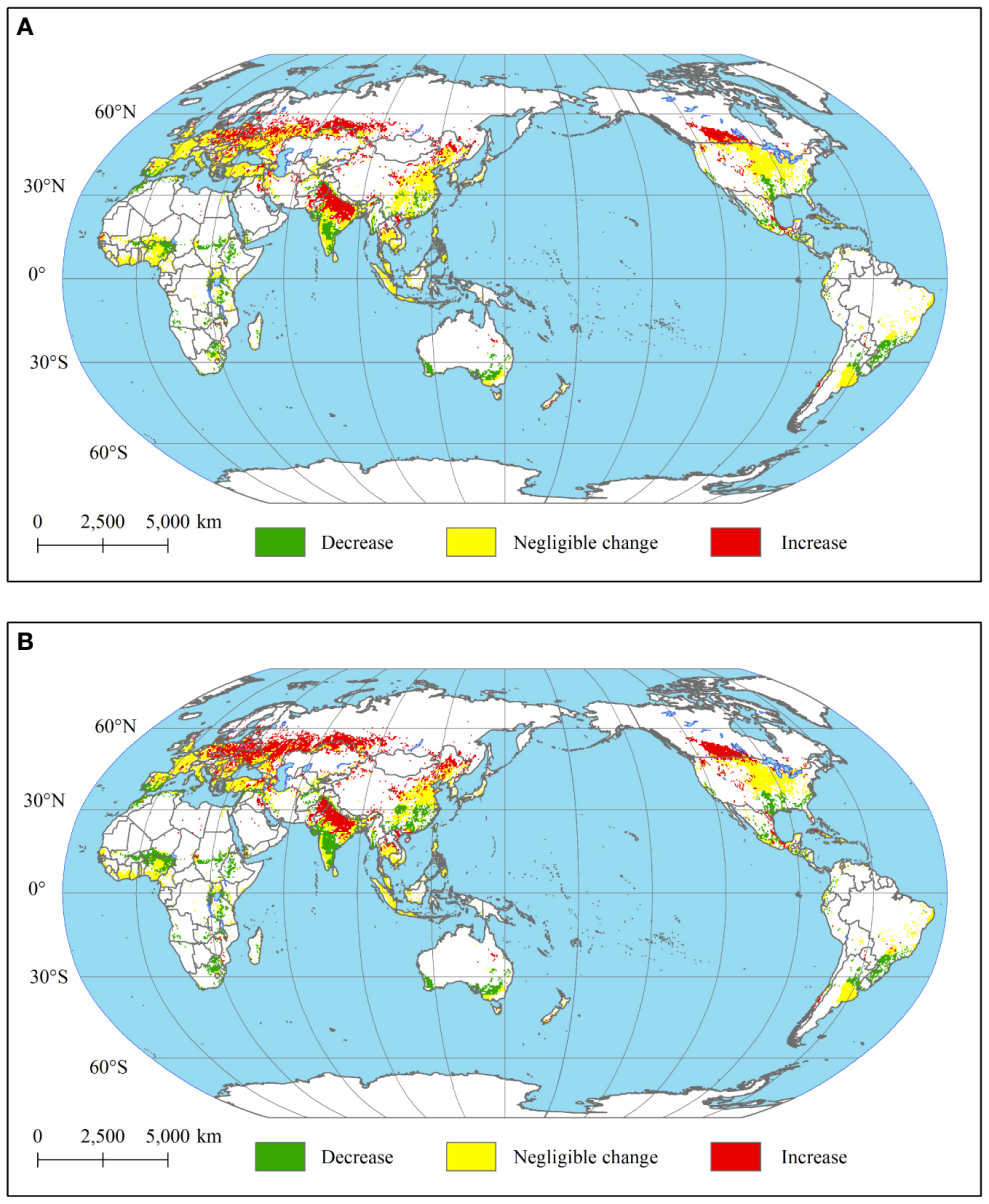
Changes in suitability for wheat planting [**(A)** difference in suitability between the 1.5°C warming target and baseline, **(B)** difference in suitability between the 2°C warming target and baseline].

Specifically, over 85% of the suitability increase occurred within areas that are highly suitable or moderately suitable for wheat cultivation, while more than 41% of the decrease in suitability is observed in regions that are initially classified as marginally suitable or unsuitable for wheat cultivation (**Supplementary Figure S1.4**). According to **Figure 4**, the region where suitability increase is mainly located in the colder regions of the major wheat-growing countries/regions, such as Russia, Canada, northeastern Western Europe, north-western China, northern India, Pakistan, and Iran. The negative effects of warmer temperatures on wheat cultivation will predominantly be experienced at low latitudes. Specifically, Africa, southern Australia, southern Brazil, northern Argentina, southern China and the southern United States will witness a decline in wheat planting suitability (**Figure 4**). The aforementioned trends imply that the polarization of wheat planting suitability may be exacerbated by climate change. The major regions for wheat production will become more suitable for wheat planting under global warming. In contrast, achieving self-sufficiency in wheat is increasingly challenging for less-developed and low-latitude regions. We further discussed these notable findings in Section 4.2 with support of additional meterials.

### 3.3 The reliability of the wheat planting suitability prediction

**Figure 5:**
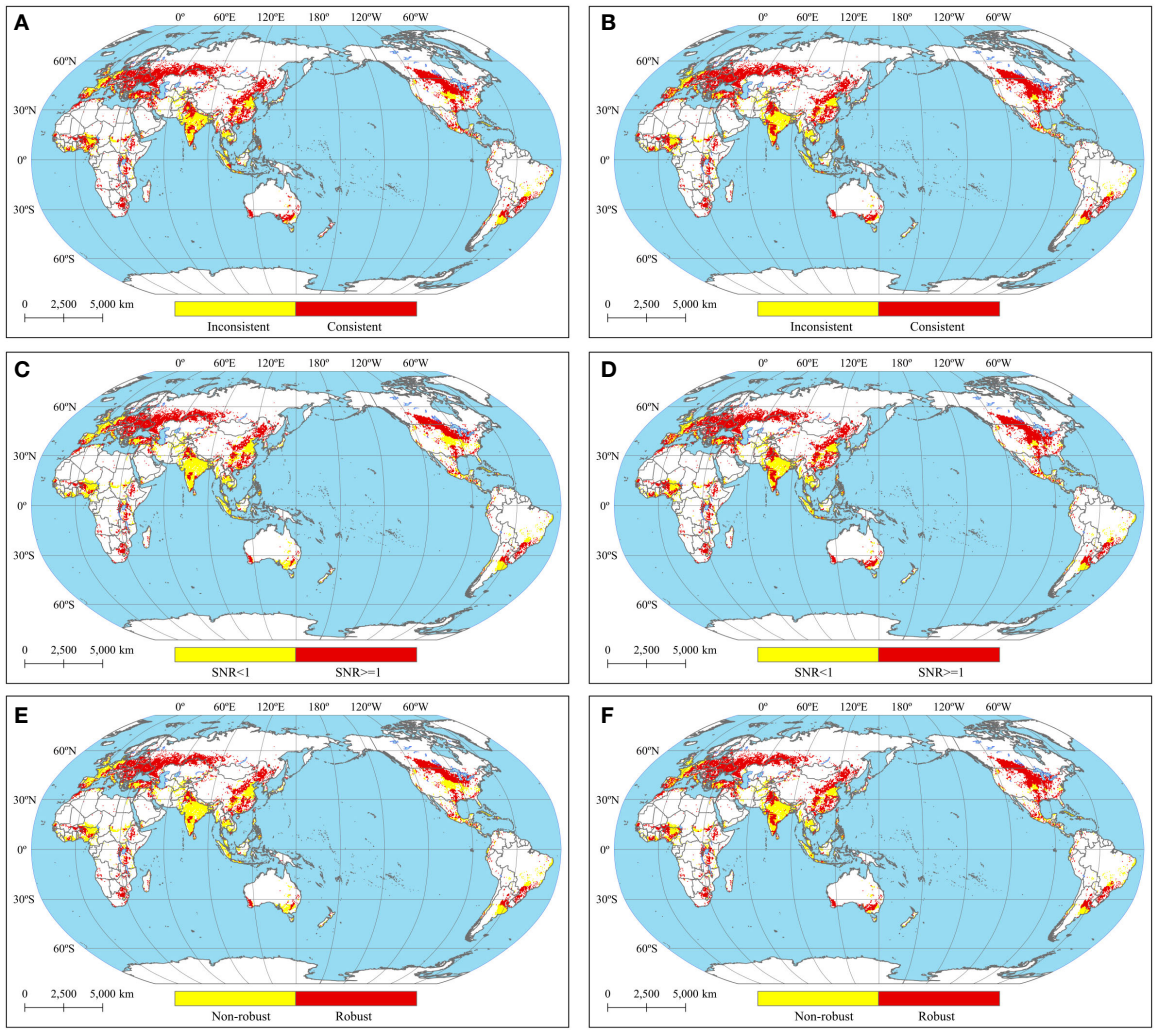
The robustness of the change in suitability for wheat planting under climate change [**(A)** the model agreement for the change in suitability under the 1.5°C target compared to the baseline period, **(B)** the same as **(A)** but for the 2°C target.**(C, D)** The same as **(A, B)** but for the SNR. **(E, F)** the same as **(A, B)** but for the total robustness[.

**Figure 5** shows the robustness of the potential wheat planting suitability under the multimodal ensemble. The percentages of areas with consistent signals of wheat potential planting suitability were 70% and 77% at targets of 1.5°C and 2°C, respectively. These proportions were significantly greater than those with inconsistent signals. Therefore, it can be concluded that most regions of the world experienced consistent changes in wheat potential planting suitability under the multimodal ensemble. For the 1.5°C and 2°C targets, the proportions of areas with a signal-to-noise ratio (SNR) greater than 1 were 58% and 68%, respectively. The potential planting suitability of wheat changed significantly under each model, with considerable variation and some uncertainty. Furthermore, the proportions of areas meeting the two indicators, i.e., the robustness of the change in wheat planting suitability, were 58% and 68% under the 1.5°C and 2°C targets, respectively.

The regions showing variations in consistency and SNR robustness of changes in potential wheat planting suitability at the two targets were primarily the east-central United States, western Europe, central Africa, south-central India, east-central United States, Australia, Brazil and Argentina. Wheat cropping suitability in these regions was found to be stable only at the 2°C target. This suggests that the potential for wheat cropping suitability in these regions will be further enhanced by climate change at the 1.5°C to 2°C target. However, further research is needed to assess the impact of global warming on wheat planting suitability in these regions, as the change in wheat suitability for these regions at the 1.5°C target is more uncertain among models.

**Supplementary Figures S1.5**, **S1.6** show the probability density and cumulative probability distribution of potential wheat suitability changes. Potential wheat suitability changes followed a normal distribution, with peak values of approximately 0, implying small suitability changes in most regions. At the 1.5°C target, the peaks were larger, indicating greater suitability changes than those at the 2°C target. At the 2°C target, the differences between the models were smaller than those at the 1.5°C target, with the largest changes in potential wheat suitability in the HAD model. Overall, at the 2°C target, the HAD model data showed the greatest changes in global wheat potential suitability, creating uncertainty.

The Friedman test revealed that the models differed in potential wheat planting suitability changes at the same warming target. Based on the robustness analyses, this difference may stem from the large SNR variations in individual wheat planting suitability changes under each climate model. The MIR model showed a larger difference from the others, with a smaller suitability change, possibly due to its lower sensitivity to climate warming. Notably, SNR is significantly correlated with the resilience of changes in potential wheat suitability, which was discussed in detail in Section 4.3.

## 4 Discussion

### 4.1 Global warming would favour wheat planting in more regions

**Figure 6:**
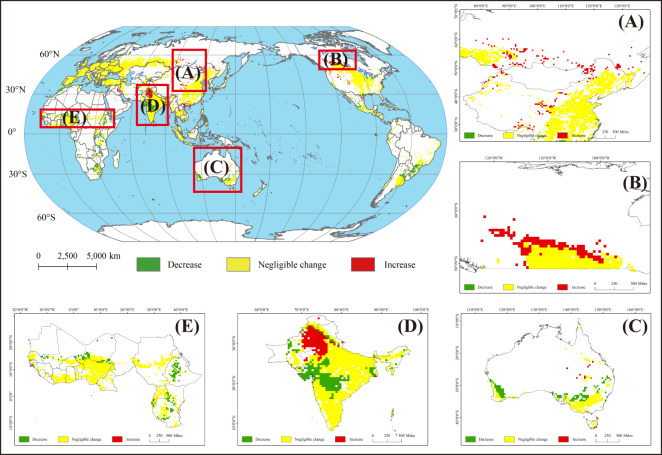
Change in the wheat cultivation suitability between 1.5°C and 2°C global warming target. **(A)** China and Russia, **(B)** Canada, **(C)** Australia, **(D)** India and Pakistan, **(E)** Essential wheat-producing countries in Africa.

Contrary to initial expectations, no substantial evidence is found indicating that further climate warming would significantly diminish global wheat suitability. When global temperatures increase to 2°C, 10% of the global area will become more suitable for wheat cultivation compared to the 1.5°C warming target, while only 6% of the area will experience a decrease in wheat cultivation suitability (**Supplementary Table S2.5**). Overall, the areas of change are mainly located at the edges of the global wheat-growing region (**Figure 6**). Wheat planting suitability rise in the region is mainly concentrated in major wheat-producing countries in the northern hemisphere, and showed a tendency to spread to the Arctic region. Conversely, regions near the equator and the southern hemisphere countries face significant challenges due to rising temperatures impacting wheat cultivation. This suggests that a change in global warming from 1.5°C to 2°C will promote wheat cultivation in cold regions while amplify the adverse effects of temperature raising on wheat cultivation in warming area.

Furthermore, with a rise in temperatures from 1.5°C to 2°C global warming targets, planting suitability will increase even more significantly in the northern regions of major wheat producing nations such as Russia, China (**Figure 6A**) and Canada (**Figure 6B**) compared to a 1.5°C warming target. This presents promising implications for global wheat supplies in the future. The raising temperatures leads to a reduction in the duration of the overwintering period, while simultaneously extending the effective growth phase of wheat and enhancing bioaccumulation (Du et al., 2021; He et al., 2020). These changes are expected to promote enhanced growth and resilience. For main wheat-producing countries in the mid-latitudes, the impact of rising temperatures on the wheat planting suitability cannot be generalised. In India and Pakistan, the trend is basically a decline in the suitability of planting in the south and an increase in the suitability of planting in the north (**Figure 6D**). The situation is reversed for countries in the southern hemisphere, such as Australia (**Figure 6C**). However, in the low-latitudes regions that are less-developed and heavily rely on imported wheat, higher temperatures appear to pose challenges for wheat cultivation. For instance, essential wheat producing countries in Africa (e.g., Ethiopia) will become less suitable for this crop (**Figure 6E**).

Our result aligns with prior researches. For instance, Yue et al. (2019) indicated that climate change is likely to have a positive influence on wheat cultivation in mid-high latitudes. Similarly, Ortiz et al. (2008) suggested that climate change might lead to wheat cultivation zones in Europe and North America expanding up to 65°N. Sun et al. (2015) presented winter wheat producing region of China is also expected to move northward. However, our findings contradict the study by Guo et al. (2024), which suggests that wheat-producing countries such as Western Europe, Canada, and the United States may experience a decrease in future wheat demand due to population decline. As a result of supply and demand considerations, local farmers might reduce the cultivation area for wheat by the end of this century.

It is important to emphasize that our analysis only addresses climate change impacts on wheat planting suitability. In fact, global climate change can also impact the global security of wheat production in various ways. For instance, high temperature will be detrimental to the photosynthesis, growth and development and pollen fertility of wheat (Grant et al., 2011; Eyshi Rezaei et al., 2015). At the same time, due to the reduced transpiration efficiency of vegetation by high temperature (Lobell et al., 2015), surface evaporation is significantly increased, thereby exacerbating the severity of drought (Dai, 2013; He and Zhou, 2016) and causing more severe compound dry hot extremes. Therefore, in addition to investigating the suitability of wheat cultivation, future research is needed to study the impact of high temperatures and compound dry-hot events on wheat production.

### 4.2 Global warming exacerbates the imbalance between wheat supply and demand regions

Based on Food and Agriculture Organization (FAO) data, the wheat yield of China, the United States, Russia, Ukraine, Canada, Australia, India, Pakistan, Turkey, and Western Europe collectively represents more than 80% of the world’s wheat production (FAO, 2021). Hence, we chose these nations to investigate the consequences of different targets on future wheat cultivation.

The study unveiled that global warming exerted positive influences on wheat cultivation in elevated latitudes regions. Under 1.5°C warming target, the suitability of wheat in West Europe, Pakistan, Northern India, Russia, Canada and northern China improved compared with baseline (**Figure 4A**). Moreover, the 2°C warming target was more favourable for wheat cultivation in Russia, Canada and northern China (**Figure 4B**). Specifically, in Canada, the 2°C warming led to a significant increase in the suitability for wheat cultivation across almost all northern regions that were previously characterized as marginally suitable for wheat planting (**Figure 3**). On the other hand, our study found that the suitability of growing wheat in Indian and Pakistani regions varied very drastically under two warming targets. The impact of global warming on wheat cultivation suitability in southern and northern India exhibited distinct patterns. In northern India, higher temperatures were found to enhance the suitability for wheat cultivation, whereas in southern India, they were observed to suppress it. In contrast, regional suitability in Pakistan was significantly enhanced under different temperature rise scenarios, with high crop suitability in Pakistan increasing from 2% to 13% and 22% under the 1.5°C and 2°C global warming targets, respectively (**Figure 4**). The study by Pervez et al. (2014) showed that climate change, including global warming, will not have a negative impact on wheat production in Pakistan, while the study by Rehana et al. (2012) further showed that climate change has a positive impact on wheat production. This suggests that climate change may contribute to making Pakistan a new growth point in the global wheat production chain.

While wheat cultivation thrives at cold regions in mid-high latitudes, warmer temperatures have negative impacts on southeastern Australia, southeastern China, southern America, southern Brazil and Africa countries (**Figure 4**). In particular, the warming scenario reduces Australia’s suitability for wheat from 97% to 93% and 92% of moderately suitable regions at 1.5°C and 2°C targets, respectively. Similarly, rising temperatures have decreased the wheat planting suitability in wheat-import dependence regions, such as Africa countries (**Figure 6E**). For instance, at 1.5°C and 2°C targets, Ethiopia encounters a decline in agricultural suitability across 90% of its regions, with 1% and 3% of the area transitioning from marginally suitable for wheat planting to unsuitable, respectively.

This phenomenon is likely to further exacerbate the imbalance between global wheat supply and demand. As major wheat-producing regions, such as Western Europe, Canada and Russia, will become more suitable for wheat cultivation under global warming, which will consolidate the position of these countries or regions as wheat-exporting regions. In contrast, achieving self-sufficiency in wheat is increasingly challenging for less-developed and low-latitude regions, particularly African countries, and the reliance of African nations on wheat imports is projected to escalate further. Currently, the wheat supply in Africa heavily relies on imports (Negassa et al., 2013; Shiferaw et al., 2013), The market demand and cost for wheat in Africa are steadily increasing year after year (Tadele, 2017). As global warming becomes an indisputable fact, the suitability of wheat cultivation in these regions would be further reduced (**Figure 6E**), which will further dampen the interest of local farmers in wheat cultivation. Zhang et al. (2022) also presented that the profitability of farmers in advanced economies can be maintained or even raised, but this will inevitably cause economic losses and inequalities for farmers in less-developed, wheat-importing countries with global warming.

In light of the challenges posed by global warming, particularly in African countries, intensified efforts are imperative to achieve wheat self-sufficiency. For example, the agricultural sector could employ management strategies (Li et al., 2024), such as adjusting sowing dates (Xiao et al., 2020), optimizing irrigation practices (Shrestha, 2014), and enhancing fertilization strategies (Yu et al., 2022) to effectively mitigate the impacts of global warming. Furthermore, policymakers in these regions may need to actively introduce and breed wheat varieties that are resistant to high temperatures, as well as strengthening international education and training on advanced agronomic techniques to minimize the adverse effects of climate change (Hawkesford et al., 2013; Liu et al., 2018).

### 4.3 SNR is significantly associated with the robustness of changes in suitability

Xiong et al. (2020) proved that the choice of GCMs is one of the essential factors affecting the uncertainty in predicted responses of yield to warming. This study also found that the differences between the two targets and GCMs caused uncertainty in the influence of climate warming on the potential suitability of wheat cultivation. Compared to a 1.5°C target, under a 2°C target, there is a marked increase in the area of regions with robust suitability changes affected by climate warming, which indicates that a further 0.5°C warming will result in a more significant impact on the potential planting distribution of wheat. Furthermore, at the 2°C target, a robust but slight increase in wheat planting suitability is expected over Western Europe and the central and eastern United States, and a robust but significant decrease is expected over south-central India, Australia and Argentina. At the two targets, the potential suitability of wheat cultivation in France, India, the Jiao Dong Peninsula in China and Argentina is not as robust as we expected. Therefore, the impact of global warming targets on the potential suitability of wheat cultivation in these areas may need further investigation. Meanwhile, by analysing the differences in the potential wheat planting suitability among various climate scenarios, it can be found that the MIR climate scenarios may have larger differences from other model data, and the choice of this climate scenarios may introduce noise to the robustness of the results.

We then analysed the relation between the extent of the dynamic variation in wheat cropping potential under the two climate change scenarios in contrast with the baseline period and the corresponding resilience. First, the magnitude of change in wheat planting suitability (change), the number of models with consistent signals of change in wheat planting suitability (consistency), the signal-to-noise ratio (SNR), and the robustness of change in wheat planting suitability (robustness) were statistically determined for each grid cell; then, based on the above statistical data, we analysed the relationship between the correlation of change with consistency, SNR, and robustness. The results showed that change was positively correlated with SNR under the 1.5°C target, while change was negatively correlated with robustness when the effects of consistency and SNR were removed. Under the 2°C target, removing the effects of consistency and SNR revealed no correlation between change and robustness. The true correlation remained between change and SNR, both of which showed a positive correlation.

In conclusion, at the two targets, the magnitude of change in potential wheat planting suitability (change) was positively correlated with SNR. This means that the greater the magnitude of change in wheat planting suitability was, the more valid information was obtained. However, the magnitude of change in potential wheat planting suitability was only negatively correlated with the robustness of change at 1.5°C target. In other words, the more drastic the change in wheat suitability was, the less robust it was. The results above indicate that the SNR is significantly associated with the robustness of changes in potential wheat suitability.

### 4.4 Method viability and upcoming research

In this study, the Maxent model was applied to quantify the planting suitability of wheat at the 1.5°C and 2°C targets based on five GCMs. We also analysed the impact of different types of global warming on wheat cultivation and the reliability of these results. The statistical and spatial accuracy confirmed that the Maxent model can recognize the potential wheat planting suitability. In the baseline and two warming targets, the spatial distribution patterns of the levels of wheat planting suitability in the two targets are roughly the same, and the highly suitable area mostly appears in Pakistan and India. The land that is suitable for wheat cultivation makes up the largest part, and its distribution range is also the widest. The regions with moderate and high suitability for future wheat cultivation, as projected by the Maxent model, closely align with the primary wheat-producing nations according to FAO statistical records (FAO, 2021). In addition, the area proportion of marginal suitability and unsuitability for planting wheat is small. As studies have shown that the main products in these areas are indeed not wheat (Leff et al., 2004; Monfreda et al., 2008), it can be considered that these areas are very unlikely to grow wheat. Therefore, the potential wheat cultivation distribution we estimated is reliable.

It should be noted that there are some limitations in our study. First, the potential wheat cultivation we estimated was driven by weather and soil factors only, while factors related to wheat growth characteristics on a larger scale were not taken into account. In addition, we did not discuss the impact of socioeconomic development and management factors, which are also critical for taking serious actions to adapt to climate change. Socio-economic factors such as revenue (Guo et al., 2024) and market demand (Su et al., 2023) will significantly change farmers’ planting decisions, which in turn will affect the distribution of crop cultivation. Similarly, the international situation also brings some uncertainty to the distribution of wheat cultivation, e.g., wars may lead to abandonment of cultivated land or reclamation of cultivated land in non-war zones, and unrest in wheat-exporting countries may have a global impact through the trade chain (Chowdhury et al., 2023; Rawtani et al., 2022). Also, sowing and harvesting dates have shifted, crop distribution and structure have been adjusted, and agronomy and breeding have advanced (Anwar et al., 2007). Consequently, further studies are essential to determine the potential global wheat cultivation distribution driven by both socioeconomic development and climate change. Second, in addition to directly affecting the potential planting distribution of wheat, future temperature rise may also increase the frequency of climate extremes such as heatwaves and flash droughts (Sheffield and Wood, 2008; Dai, 2011, 2013; Ault et al., 2014; Trenberth et al., 2014; Lehner et al., 2017), which could potentially have adverse effects on wheat cultivation (Asseng et al., 2015; Liu et al., 2016; Yue et al., 2018). Therefore, the expected increase in extreme event impacts should be given more attention.

## 5 Conclusion

The potential global wheat distribution under 1.5°C and 2°C targets was projected using the Maxent model and 5 GCMs. The results suggest that there will be notable shifts in suitability for cultivation. Both warming targets are expected to enhance the suitability of more regions for wheat production, primarily concentrated in the cold zones of major wheat-producing countries at mid to high latitudes. Conversely, low latitude countries heavily reliant on wheat imports will face adverse impacts from climate warming on their ability to cultivate this crop. This phenomenon is likely to exacerbate the existing imbalance between global wheat supply and demand. Our research reveals the global distribution pattern of wheat cultivation under different temperature rise targets and uncovers the potential impacts of climate warming on regional variations in wheat suitability. The aforementioned statement offers valuable insights for regional and international policymakers to effectively address the pressing issue of climate change, devise comprehensive mitigation strategies, and ensure the preservation of global food security.

## Data availability statement

The datasets presented in this study can be found in online repositories. The names of the repository/repositories and accession number(s) can be found below: [Zenodo] https://doi.org/10.5281/zenodo.8354268 [DOI:10.5281/zenodo.8354268].

## Author contributions

XG: Writing – review & editing, Writing – original draft, Formal analysis, Data curation. PZ: Writing – original draft, Software, Methodology, Data curation. YY: Investigation, Funding acquisition, Writing – review & editing, Writing – original draft, Supervision, Project administration, Conceptualization.
