# Supplementary material for: Global wheat planting suitability under the 1.5°C and 2°C warming targets
Source: Front Plant Sci. 2024 Jun 17;15:1410388. doi: 10.3389/fpls.2024.1410388 (PMC11215128; doi:10.3389/fpls.2024.1410388)
Supplement: Supplementary file 1 [file DataSheet_1.docx]

Supplementary Material

# Supplementary Methods

## Environmental variable choice

Maxent model was employed to predict wheat planting suitability under climate change. This model was developed based on the principle of maximum entropy, ensuring that the result of species distribution is closest to geographic uniformity (Phillips et al., 2006). The Maxent model uses covariate data from the occurrence records and the background sample to estimate the potential distribution (Elith et al., 2011). These covariate data consist of the presence data and information on the environmental and/or spatial characteristics of those locations (Phillips et al., 2006; Phillips and Dudík, 2008).

In general, the suitability of land for cultivation is significantly influenced by environmental factors and agronomic practices such as fertilization and irrigation. However, due to the challenges in quantifying agronomic techniques and the lack of authoritative data on their effectiveness under temperature rise scenarios, this study solely focuses on estimating the suitability of wheat cultivation based on environmental variables. Therefore, the primary objective of this paper is to establish a quantitative relationship between global land suitability and environmental factors.

The growth of wheat is affected by both climate and soil (Motuma et al., 2016). Based on previous studies, we established an indicator evaluation system for wheat suitability, including climatic factors and soil factors: ≥0°C accumulated temperature, annual precipitation, annual average temperature, average temperature of the coldest month, pH, drainage, electrical conductivity, exchangeable sodium percentage, soil texture, soil depth (Mendas and Delali, 2012; Barsari et al., 2014), and slope were selected as the environmental variables to constrain the growth of wheat. The study conducted by Yue et al. (2019) successfully applied these environmental factors to accurately simulate the distribution of wheat cultivation under climate conditions.

## Training sample selection

Addressing the issue of sample bias is of utmost importance when employing the Maxent algorithm for estimating species distribution (FAO, 2021). Given the global significance and extensive cultivation of wheat, it becomes imperative to meticulously select representative occurrence points from the vast distribution dataset (Zhu et al., 2018).

To mitigate selection bias, an appropriate sample size is carefully chosen, and the samples are ensured to adhere to the requirements of random distribution. First, we compared the mainstream historical wheat distribution data, including the fraction of wheat-harvested area (FWHA) (Monfreda et al., 2008), the wheat total harvested area map using the spatial production allocation model (SPAM) (You et al., 2014), the wheat yield statistics data (FAO, 2000), and MIRCA2000 (Portmann et al., 2010). Among these data, FWHA data has high precision and reliable data sources. Monfreda et al. (2008) compiled a comprehensive global database on harvested area and yield, including FWHA, which provides detailed information on the area (harvested) and yield of 175 distinct crops worldwide in the year 2000. This database was constructed by collecting agricultural census data and survey information from the smallest political units that were reasonably obtainable for 206 countries. Therefore, we selected FWHA as the primary source for identifying occurrence points of wheat distribution.

Furthermore, to ensure the validity of our samples, we excluded FWHA data with extremely low probabilities. The original dataset contained numerous values indicating lower harvest area ratios for wheat cultivation. If we had sampled according to the distribution of FWHA alone, it would have led to generally lower predicted suitability results and an inaccurate reflection of regions with high suitability for wheat growth. According to the IPCC's probability definition, events with a probability below 1% are considered exceptionally unlikely to occur. Including these sample points could introduce selection bias and adversely affect the obtained results. Therefore, we made sure to exclude data below 0.01 from our samples.

Finally, the data distribution of FWHA samples was clustered based on the Jenks Natural Breaks (Jenks and Caspall, 1971) classification method after removing samples with very low values. This method effectively reduces variance within classes while maximizing variance between different classes. Utilizing the Jenks Natural Breaks method, we classified the valid samples into five distinct groups. The group exhibiting the highest FWHA value (indicating superior adaptation to wheat cultivation) consisted of a total of 3114 grids, which represented the smallest group size among all categories. To ensure proportional representation in our sample set, we randomly selected 3100 grids from each group, resulting in a total selection of 15,500 samples, which occupied approximately 5% of all grids.

**Reference**

Barsari, S.N., Parsmehr, M., and Kashani, S.J. (2014). Qualitative assessment of land suitability for the cultivation of irrigated wheat and barley by using simple limited and the number and limiting intensity method (case study: Esfarvarin region of qazvin province). Agricultural Advances 3, 38-47.

Elith, J., Phillips, S.J., Hastie, T., Dudík, M., and Chee, Y.E., et al. (2011). A statistical explanation of maxent for ecologists. Diversity & Distributions 17, 43-57. doi:10.1111/j.1472-4642.2010.00725.x

Food and Agriculture Organization. (2000). Global wheat statistics 2000. https://www.fao.org/faostat/

Food and Agriculture Organization. (2021). Global wheat statistics 2021. <https://www.fao.org/faostat/>

Jenks, G.F., and Caspall, F.C. (1971). Error on choroplethic maps: definition, measurement, reduction. Annals of the Association of American Geographers 61, 217-244. doi:https://doi.org/10.1111/j.1467-8306.1971.tb00779.x

Mendas, A., and Delali, A. (2012). Integration of multicriteria decision analysis in gis to develop land suitability for agriculture: application to durum wheat cultivation in the region of mleta in algeria. Comput. Electron. Agric. 83, 117-126. doi:10.1016/j.compag.2012.02.003

Mohammed, M., Suryabhagavan, K.V., and Balakrishnan, M. (2016). Land suitability analysis for wheat and sorghum crops in wogdie district, south wollo, ethiopia, using geospatial tools. Appl. Geomat. 8, 57-66. doi:10.1007/s12518-016-0168-5

Monfreda, C., Ramankutty, N., and Foley, J.A. (2008). Farming the planet: 2. Geographic distribution of crop areas, yields, physiological types, and net primary production in the year 2000. Glob. Biogeochem. Cycle 22. doi:10.1029/2007GB002947

Phillips, S.J., Anderson, R.P., and Schapire, R.E. (2006). Maximum entropy modeling of species geographic distributions. Ecol. Model. 190, 231-259. doi:10.1016/j.ecolmodel.2005.03.026

Phillips, S.J., and Dudík, M. (2008). Modeling of species distributions with maxent: new extensions and a comprehensive evaluation. Ecography (Copenhagen) 31, 161-175. doi:10.1111/j.2007.0906-7590.05203.x

Portmann, F.T., Siebert, S., and Döll, P. (2010). Mirca2000-global monthly irrigated and rainfed crop areas around the year 2000: a new high-resolution data set for agricultural and hydrological modeling. Glob. Biogeochem. Cycle 24, n/a-n/a. doi:10.1029/2008GB003435

You, L.Z., Wood, S., Wood-Sichra, U., and Wu, W.B. (2014). Generating global crop distribution maps: from census to grid. Agric. Syst. 127, 53-60. doi:10.1016/j.agsy.2014.01.002

Yue, Y., Zhang, P., and Shang, Y. (2019). The potential global distribution and dynamics of wheat under multiple climate change scenarios. Sci. Total Environ. 688, 1308-1318. doi:10.1016/j.scitotenv.2019.06.153

Zhu, A., Lu, G., Liu, J., Qin, C., and Zhou, C. (2018). Spatial prediction based on third law of geography. Ann. Gis 24, 225-240. doi:10.1080/19475683.2018.1534890

# Supplementary Figures and Tables

## Supplementary Figures


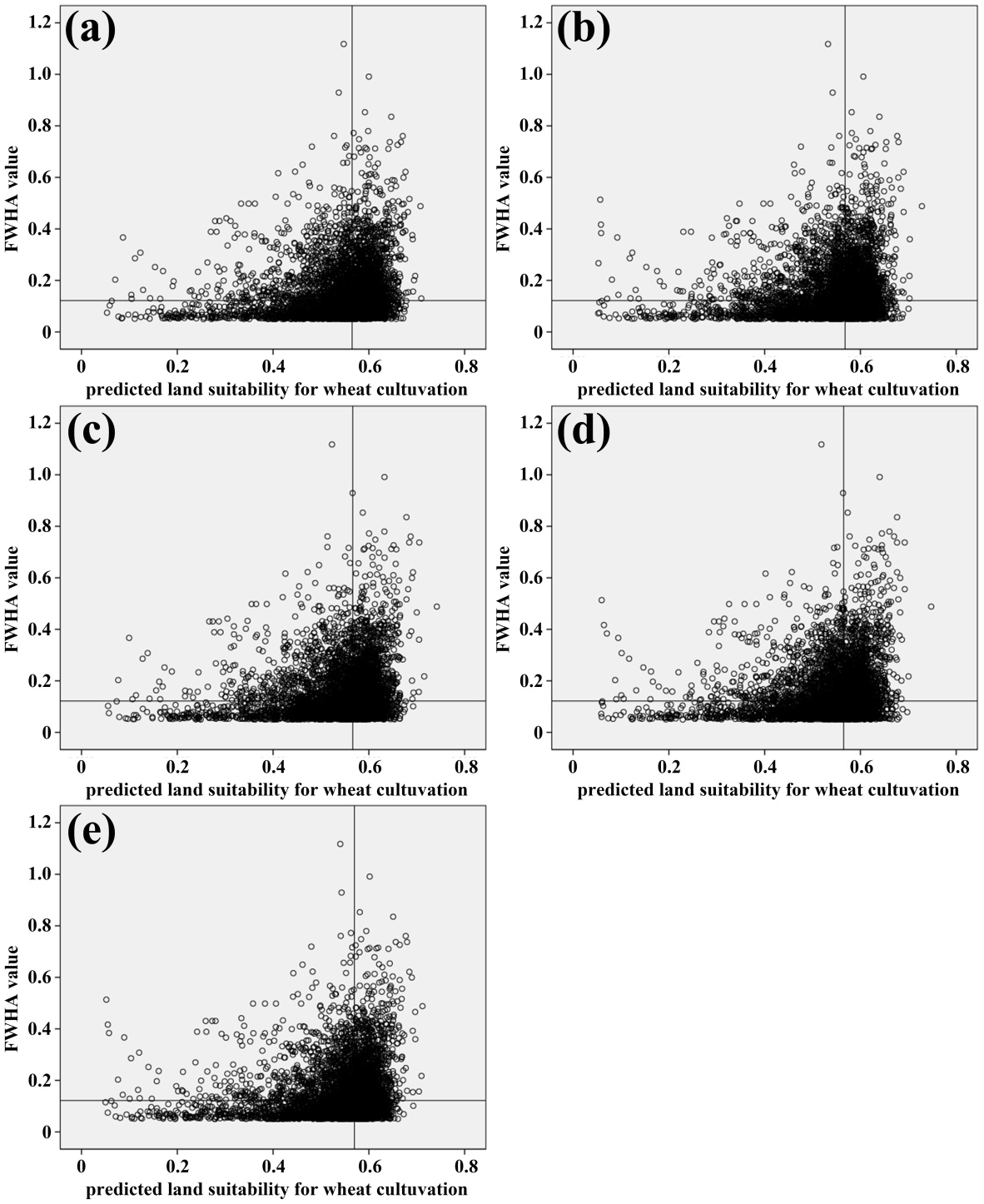


**Supplementary Figure S1.1** Scatter plot visually depicts the alignment between projected wheat planting suitability and the FWHA value using various climate models. The x-axis presents the Maxent model results, representing the likelihood of wheat presence. The y-axis portrays the FWHA value. Median values are marked by lines (a. GFDL-ESM2M, b. HadGEM2-ES, c. IPSL-CM5A-LR, d. MIROC-ESM-CHEM, e. NorESM1-M).


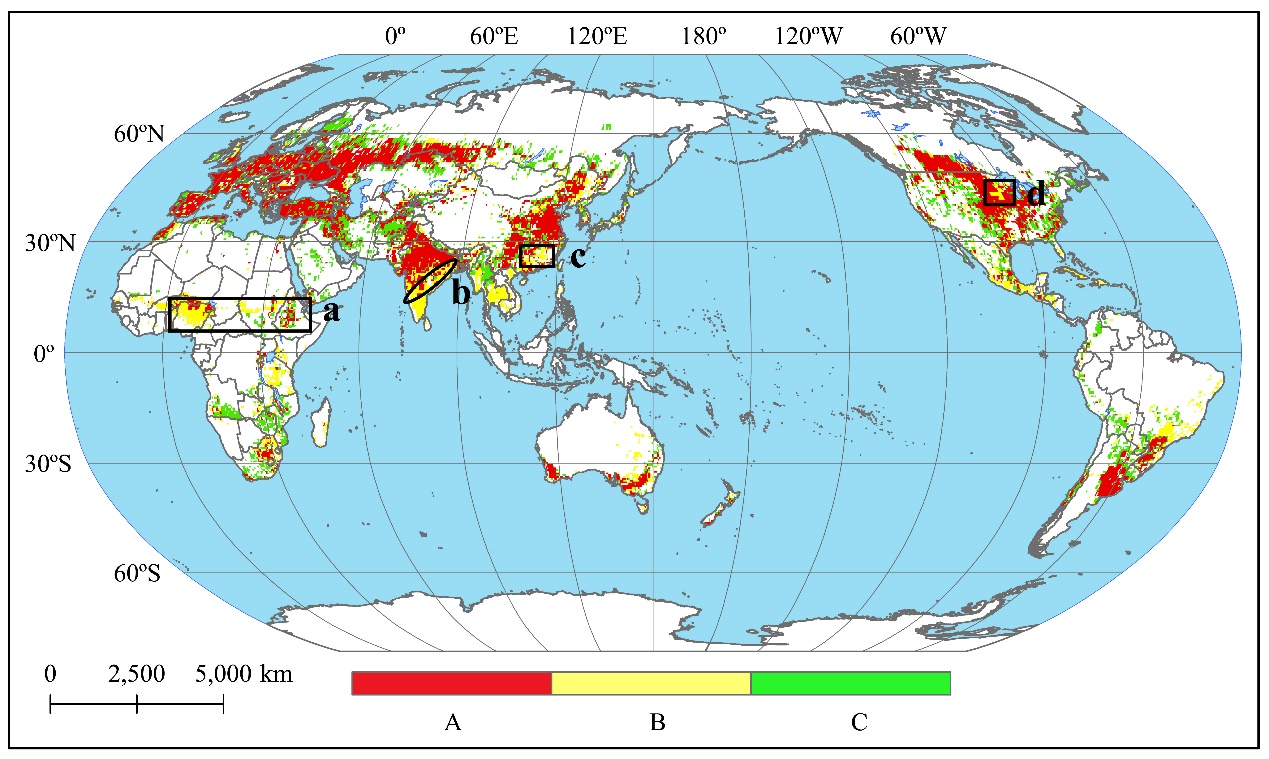


Supplementary Figure S1.2 Spatial consistency between the predicted wheat planting suitability and the SPAM (A. the region where wheat was both harvested and accurately predicted; B. the zone showed minimal or no wheat harvesting but was expected to be suitable for wheat planting; C. the region where wheat was harvested but not anticipated).


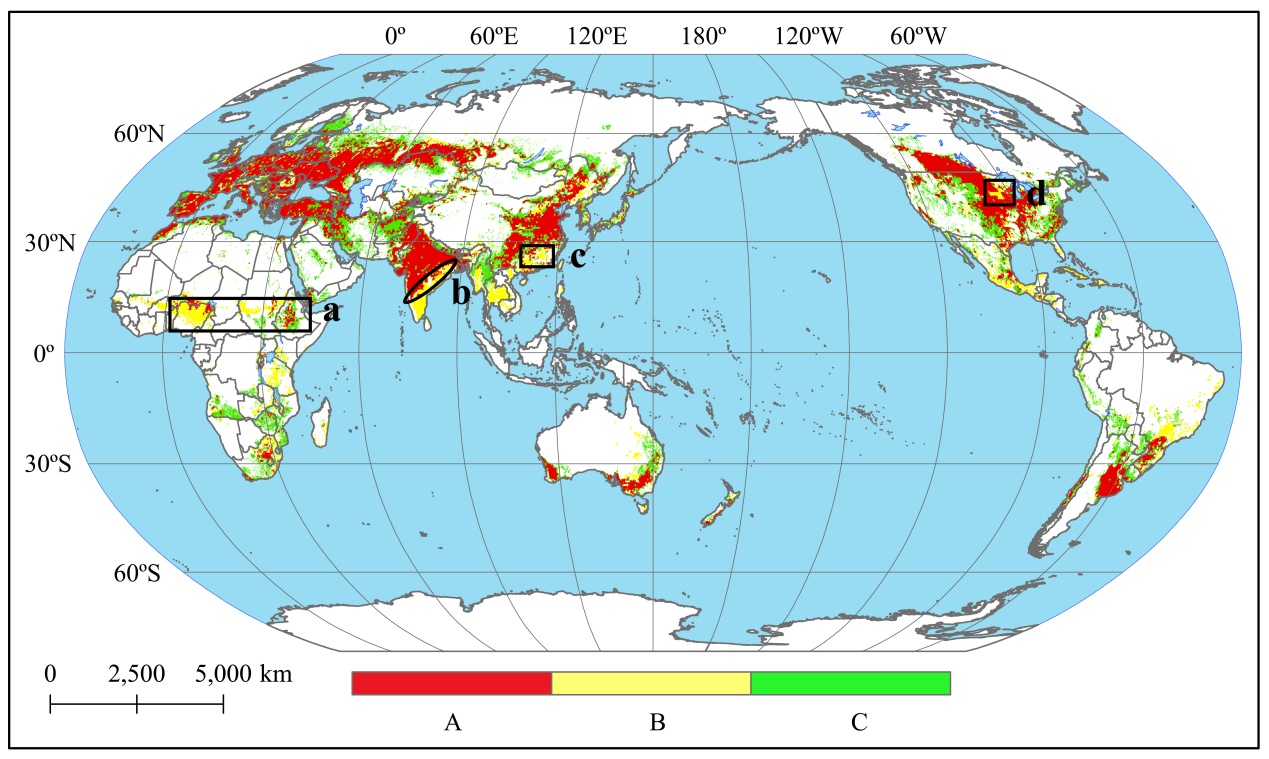


Supplementary Figure S1.3 Spatial consistency between the predicted wheat planting suitability and the integrated wheat distribution map (including FWHA and SPAM data) (A. the region where wheat was both harvested and accurately predicted; B. the zone showed minimal or no wheat harvesting but was expected to be suitable for wheat planting; C. the region where wheat was harvested but not anticipated).


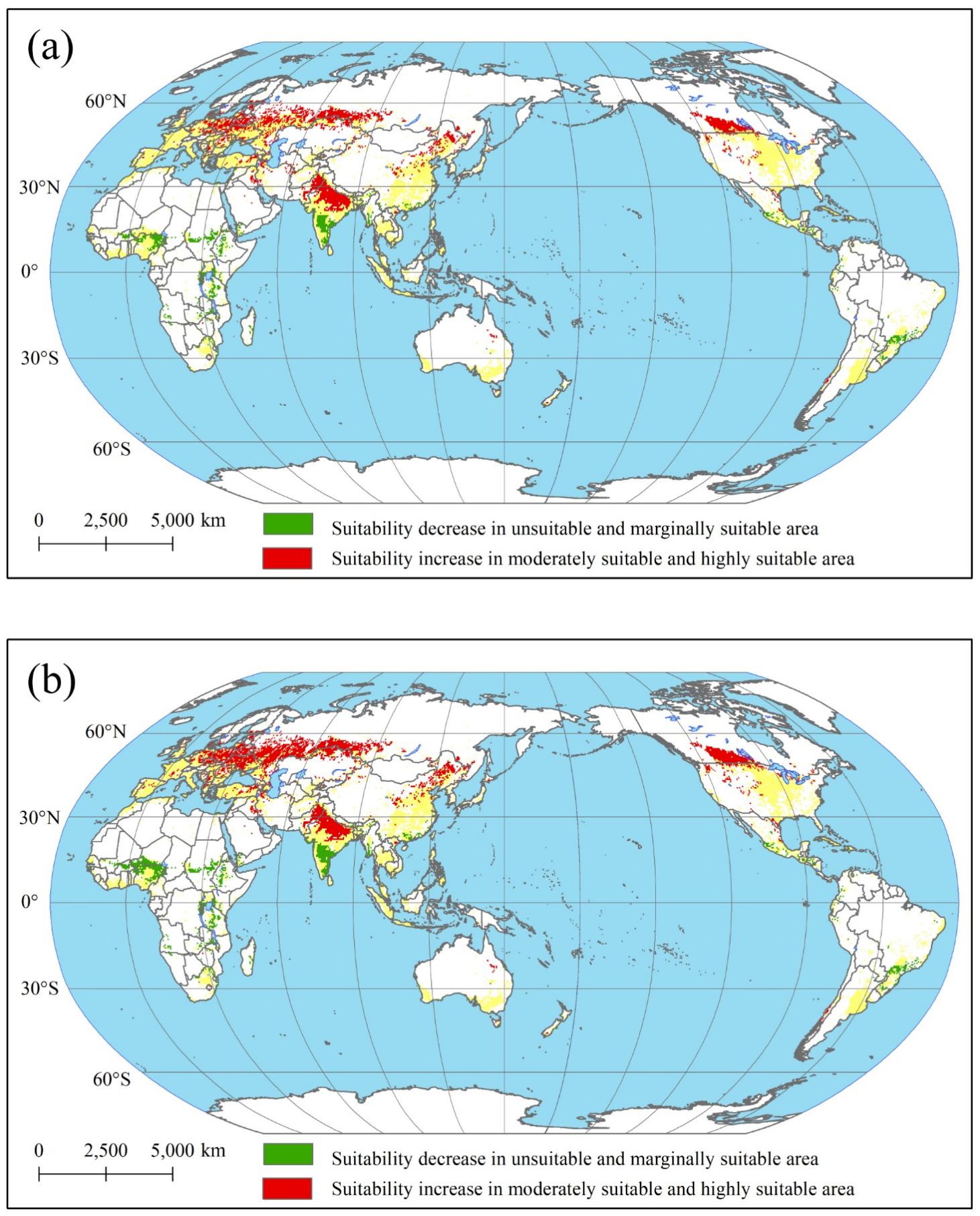


Supplementary Figure S1.4 Planting suitability changes occurring in areas with different planting suitability levels between baseline and two warming target ((a) 1.5°C warming target, (b) 2°C warming target).

**
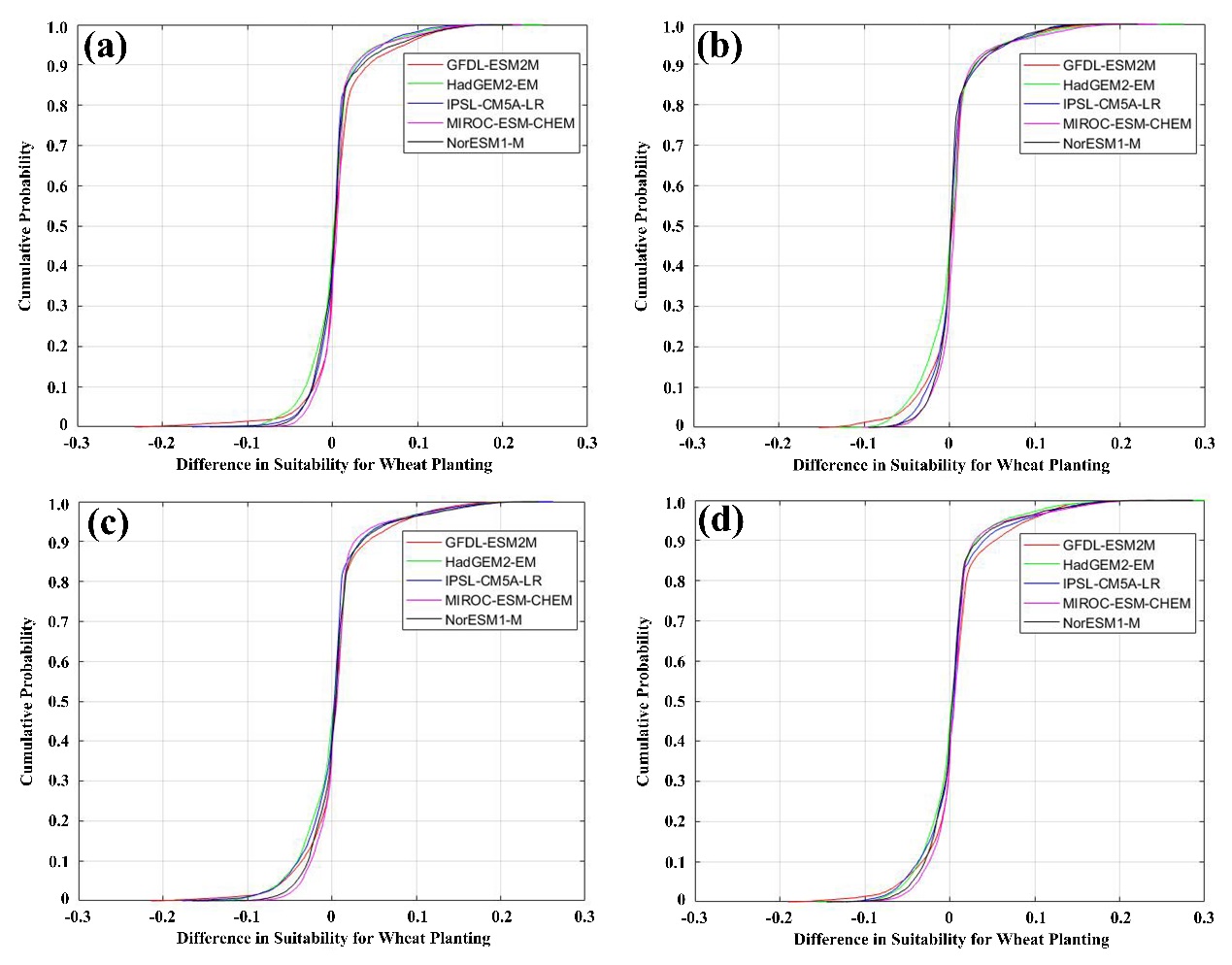
**

Supplementary Figure S1.5 Cumulative probability density function for the change in suitability for wheat planting under climate change ((a). Change in suitability under the 1.5°C warming target compared to the historical period for the RCP4.5 scenario, (b). Same as (a) but for RCP8.5, (c), (d). Same as (a) and (b) but for the 2.0°C warming target).


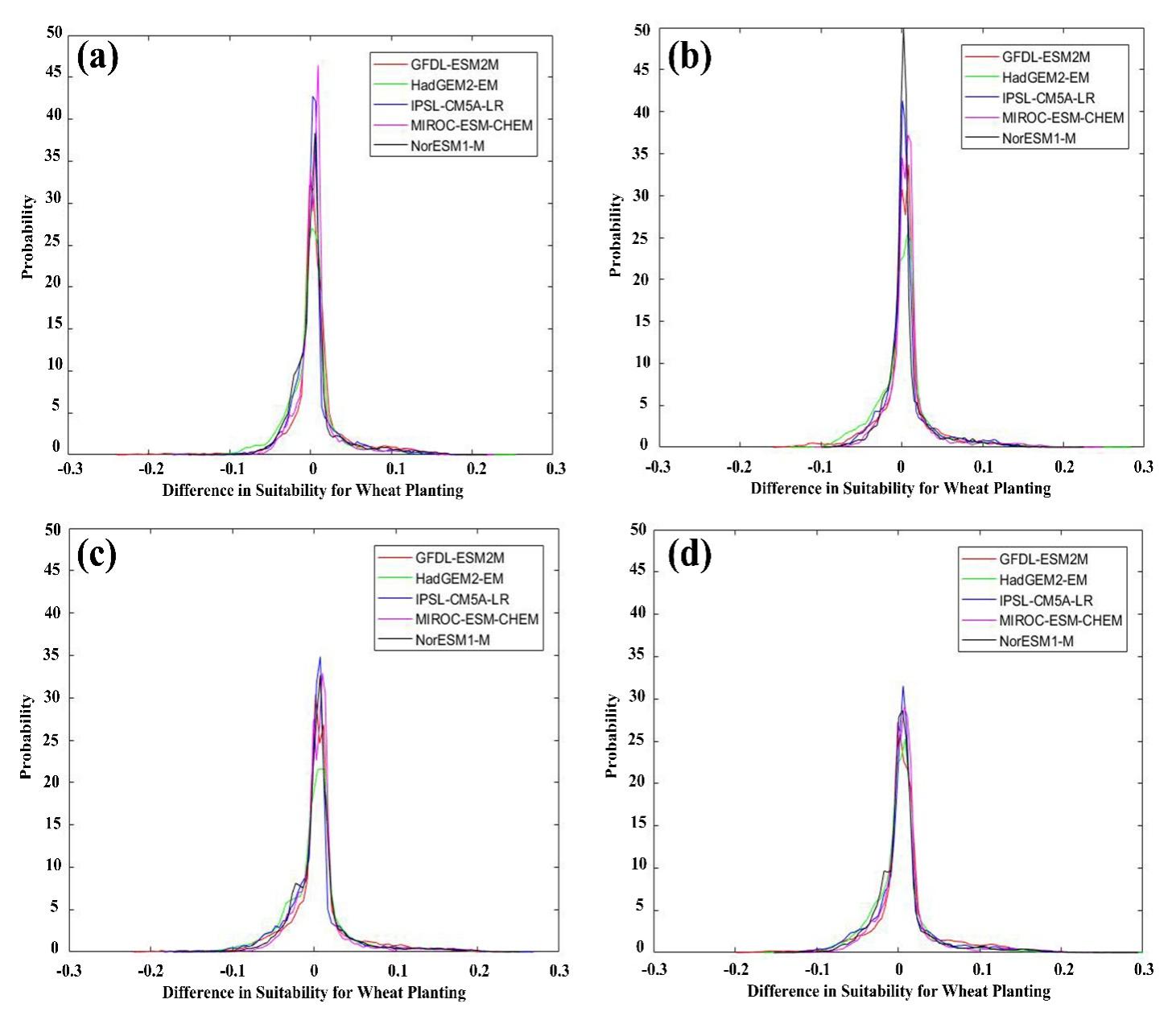


**Supplementary Figure S1.6** The probability density function for the change in suitability for wheat planting under climate change ((a). Change in suitability under the 1.5 ℃ target compared to the baseline period for the RCP4.5 scenario, (b). Same as (a) but for RCP8.5, (c), (d). Same as (a) and (b) but for the 2 ℃ target).

## Supplementary Tables

Supplementary Table S2.1 Levels of wheat planting suitability and its range.

| Suitability levels | Planting suitability range |
| --- | --- |
| Unsuitable | 0 – 0.05 |
| Marginally suitable | 0.05 – 0.33 |
| Moderately suitable | 0.33 – 0.66 |
| Highly suitable | 0.66 – 1.00 |

Supplementary Table S2.2 Levels of changed wheat planting suitability.

| Levels | Changed planting suitability range |
| --- | --- |
| Decrease | -1– -0.01 |
| Negligible change | -0.01– 0.01 |
| Increase | 0.01 – 1 |

Supplementary Table S2.3 Data sources

| Data name | Data content | Data sources and references |
| --- | --- | --- |
| ISRIC-WISE global dataset of derived soil properties | Spatial data with a resolution of 0.5° were derived from the ISRIC-WISE soil database. The soil variables include drainage, electrical conductivity of saturated paste (ECe), PH, exchangeable sodium percentage (ESP), and depth. These variables are given for the topsoil (0-30 cm) | International Soil Reference and Information Center (ISRIC) — World Soil Information  (https://www.isric.org/), Batjes et al. (2005) |
| Harmonized World Soil Database | Soil texture property with a resolution of 30 seconds by 30 seconds | FAO, International Institute for Applied Systems Analysis [IIASA], ISRIC, Institute of Soil Science – Chinese Academy of Sciences [ISS-CAS], and Joint Research Centre of the European Commission [JRC] (2012)  http://webarchive.iiasa.ac.at/Research/LUC/External-World-soil-database/ |
| Projected RCP scenario data driven by the GFDL-ESM2M, HadGEM2-ES, IPSL-CM5A-LR, MIROC-ESM-CHEM and NorESM1-M model | Global daily meteorological data at a spatial resolution of 0.5° × 0.5°, observation data (1951-2000) and projection data (2010-2099) based on the GFDL-ESM2M, HadGEM2-ES, IPSL-CM5A-LR, MIROC-ESM-CHEM and NorESM1-M model of the Coupled Model Intercomparison Project phase 5 (CMIP5) | the Inter-Sectoral Impact Model Intercomparison Project (ISI-MIP) (https://www.isimip.org/outputdata/), Hempel, Frieler, Warszawski and Piontek (2013) |
| Global Agro-Ecological Zones dataset-slope | Global terrain slope compiled using elevation data from the Shuttle Radar Topography Mission (SRTM) at a spatial resolution of 5 minutes | IIASA and FAO (2012)  http://www.gaez.iiasa.ac.at/w/ctrl?_flow=Vwr&_view=Type&idAS=0&idFS=0&fieldmain=main_lr_ter&idPS=0 |
| Global harvested area fractional for wheat | Datasets created by combining national-, state-, and county-level census statistics with a global dataset of croplands on a 5 minute by 5 minute resolution | Monfreda et al. (2008); http://www.earthstat.org/data-download/ |
| Wheat total harvested area map | Wheat harvested area established using spatial production allocation model (SPAM) with a 10 x 10 km grid-cell resolution | You et al. (2014)  http://mapspam.info |
| Land use-cropland | Land cover in 2000, 5-minute resolution | Ellis, Klein Goldewijk, Siebert, Lightman and Ramankutty (2010)  http://www.ecotope.org/products/datasets/ |
| Crop yield data | The global wheat production of various countries from 1991 to 2018 | Food and Agriculture Organization of the United Nations (FAO)  http://www.fao.org/faostat/en/#data |

**References**

[dataset] Batjes, N. H. (2005). ISRIC-WISE global data set of derived soil properties on a 0.5 by 0.5-degree grid (Version 3.0). Report 2005/08 (with data set). Wageningen: ISRIC-World Soil Information.

[dataset] Food and Agriculture Organization, International Institute for Applied Systems Analysis, International Soil Reference and Information Center, Institute of Soil Science – Chinese Academy of Sciences, & Joint Research Centre of the European Commission (2012). *Harmonized World Soil Database (version 1.2)*. Rome, Italy: FAO; Laxenburg, Austria: IIASA.

[dataset] Hempel, S., Frieler, K., Warszawski, L., Schewe, J., & Piontek, F. (2013). A trend-preserving bias correction - the ISI-MIP approach, earth system. Earth System Dynamics, 4, 219-236.

[dataset] International Institute for Applied Systems Analysis/Food and Agriculture Organization (2012). Global agro-ecological Zones (GAEZ v3.0). Laxenburg, Austria: IIASA; Rome, Italy: Food and Agriculture Organization.

[dataset] Ellis, E. C., Klein Goldewijk, K., Siebert, S., Lightman, D., & Ramankutty, N. (2010). Anthropogenic transformation of the biomes, 1700 to 2000. Global Ecology and Biogeography, 19, 589-606.

[dataset] Murakami, D. and Yamagata, Y. (2016) Estimation of gridded population and GDP scenarios with spatially explicit statistical downscaling, ArXiv, 1610.09041, URL: https://arxiv.org/abs/1610.09041.

Supplementary Table S2.4 Area proportion of different suitability levels for baseline, 1.5°C and 2°C warming targets.

|  | Total Area  (km^2^) | Unsuitable (%) | Marginally Suitable (%) | Moderately Suitable (%) | Highly Suitable (%) |
| --- | --- | --- | --- | --- | --- |
| Baseline | 27939466 | 9.17 | 24.30 | 65.89 | 0.64 |
| 1.5°C | 27939470 | 9.73 | 23.86 | 65.31 | 1.10 |
| 2.0°C | 27939471 | 9.91 | 24.05 | 64.79 | 1.25 |

Supplementary Table S2.5 Area proportion of different change levels for wheat planting suitability among different periods for the same scenario.

| Period | Decrease (%) | No significant change (%) | Increase (%) |
| --- | --- | --- | --- |
| SR1.5-Baseline | 18.90 | 53.42 | 27.68 |
| SR 2.0-Baseline | 23.79 | 42.83 | 33.38 |
| SR2.0-SR1.5 | 9.60 | 84.36 | 6.04 |

Note: The suitability increases (0.01 – 1), the suitability does not change significantly (-0.01 – 0.01), and the suitability decreases (-1 – -0.01).
